# The Power of Choice: A Study Protocol on How Identity Leadership Fosters Commitment Toward the Organization

**DOI:** 10.3389/fpsyg.2018.01677

**Published:** 2018-09-06

**Authors:** Mafalda F. Mascarenhas, Felix Dübbers, Magdalena Hoszowska, Aylin Köseoğlu, Ralitsa Karakasheva, Ayse B. Topal, David Izydorczyk, Jérémy E. Lemoine

**Affiliations:** ^1^ISPA Instituto Universitário, Lisbon, Portugal; ^2^Department of Psychology, Maastricht University, Maastricht, Netherlands; ^3^University of Social Sciences and Humanities, Warsaw, Poland; ^4^Faculty of Social Sciences, Özyeğin University, Istanbul, Turkey; ^5^Department Neuroscience, Psychology and Behaviour, University of Leicester, Leicester, United Kingdom; ^6^Faculty of Arts and Social Sciences, Sabancı University, Istanbul, Turkey; ^7^Faculty of Human Sciences, Psychology Department, University of Cologne, Cologne, Germany; ^8^ESCP Europe Business School, London, United Kingdom; ^9^C2S, Laboratory of Psychology: ‘Cognition, Health, Socialization’, University of Reims Champagne-Ardenne, Reims, France

**Keywords:** identity leadership, organizational commitment, participation in decision making, collective efficacy, team identification, SEM

## Abstract

Identity leadership (IL) describes that the effectiveness of a leader will depend upon his capacity to represent a given group, to make the group go forward, to create a group identity, and to make the group matter. An identity leader may increase commitment among his followers by increasing the perception of shared identity and giving more weight in the decision process to his followers. We aim to explore the mechanisms through which a leader who creates a shared group identity can increase organizational commitment. In the first study, we plan to conduct a cross-cultural correlational study where we aim to test if the relationship between IL and organizational commitment is mediated by team identification and mediated-moderated by participation in decision making (PDM) and collective efficacy. In the second study, we aim to explore the direction of the causality between IL and PDM. To test this hypothesis, we will conduct an experimental study in which (1) we will manipulate IL to test its influence on the perception of PDM and (2) we will manipulate PDM to test its influence on the perception of IL. Thus, we will be able to identify the role of IL and the perception of PDM on organizational commitment.

## Introduction

Leadership research in psychology theorizes about what makes successful leaders attract and bind their followers as well as keeping them committed to their goals. Numerous theories behind leadership have evolved through very different paths (Day et al., 2014). At the very beginning of organizational psychology, it was common sense that someone was either born as a leader, or not, and that there was only one effective leadership style (Day et al., 2014). The task of the born leaders was to tell followers in an effective way what to do (Durue et al., 2011). More behavioral approaches then alleged the contrary; leaders are made instead of born. It was proposed that there are characteristic traits which make you a good leader and these traits can be defined, measured, and taught so that theoretically, everybody could become a leader (Blake and Mouton, 1979). Overall those theories exclusively focused on the characteristics of the leader and did not take into account the relationship between a leader and his followers.

More contemporary theories focused on this relationship between the leader and his followers. Authentic leadership theory focuses on leaders who have an honest relationship with their subordinates and are self-aware of their goals and aspirations. There is a focus on the value they give to their subordinates and their input (Gardner et al., 2011). In the charismatic leadership theory, the leader instigates followers by his innate charisma which is attributed to the leader based on the displayed behavior (Conger and Kanungo, 1988). Regarding the relationship between leader and follower, transformational leadership theory (Judge and Piccolo, 2004) proposes that a leader can exert influence by activating and serving higher order needs in his followers. Transformational leaders guide by vision, inspire their followers, and support them in personal growth (Judge and Piccolo, 2004). Similarly, in the leader–member-exchange theory, leaders and members influence each other within a dyadic bond which is built on trust and respect (Graen and Uhl-Bien, 1995). The follower and leader often develop even an emotional relationship and support each other (Graen and Uhl-Bien, 1995).

In all of the more recent leadership theories, the goal was to extend the leader’s behavior toward the relationship between the leader and a single member. Indeed, one of the criticisms to the leadership literature is its focus on dyadic relationships (e.g., Yukl, 1999) while ignoring group level processes and the dynamic relationship between leaders and their teams (Hunter et al., 2007). Furthermore, a major part of social interaction has not been included in leadership theories so far: the relationship between a leader and his group (Gardner et al., 2010; Dinh et al., 2014). The theory of identity leadership (IL) is attempting to close that gap (Hogg, 2001) by focusing on the group identity and the group level processes that happen within a group: between the leader and his group members and between the group members themselves. Importantly, IL also differs from previous approaches by acknowledging the fact that leaders often need to create a shared sense of identity for the team to be more effective. As will be discussed in the following section, one aspect of IL (identity entrepreneurship; Haslam et al., 2011) describes that creating and shaping a shared sense of identity increases a leader’s effectiveness. Thus, IL provides guidelines on how to transform a group of people with little in common into an effective team with a shared identity, which is often not the case is newly companies.

### Identity Leadership

This new approach of leadership has appeared more recently and it perceives leadership as a group process rather than the result of leader characteristics or of a one-to-one relationship (Hogg, 2001). This model is based on the social identity theory (Tajfel and Turner, 1979) which claims that individuals have both an individual and social identity regarding the groups they belong to. Social identity theory has been used to think about processes that happen in organizational settings (Hogg and Terry, 2000). Incorporating social identity theory into the leadership literature allows for considering not only the leader, the follower, or their dyadic relationship, but the whole group and their relationship to the leader. Leadership comes as a group and a social influence process that happens within a group with a shared identity (Hogg, 2001; Hogg et al., 2012). In the first decade, social identity theory of leadership was more concerned with leader prototypicality. Empirical evidence suggested that leaders who were more prototypical of the group were more supported and more trusted by their followers (Hogg et al., 2012). Later, another model tried to identify other dimensions that enable a leader to create and maintain a social identity within its team: IL (Haslam et al., 2011). The authors defined four dimensions of IL: *identity prototypicality* – refers to being “one of us,” to be an ideal member of the group; *identity advancement* – refers to the leader’s vision for the group and his ability to make the group go forward in achieving their goals and improving their situation; *identity entrepreneurship* – the ability to create “a sense of us,” which means that the leader should be able to create a shared identity (as when politicians use “we” instead of “I” and “you”; Steffens and Haslam, 2013), and *identity impresarioship* – the ability to create moments that make the group matter. Reicher et al. (2005) described that for a leader to be efficient, it is not only necessary to create a shared social identity (i.e., a group has to exist for the leader to lead), but it is also necessary to create structures that maintain and promote the shared social identity (i.e., initiating a regular meeting to discuss group related matters and problems).

Moreover, IL leads to a better perceived performance of the leader and lower turnover intentions by followers (Steffens and Haslam, 2013). Followers are also more willing to follow and support the leader (Haslam and Platow, 2001). IL was also found to increase positive feelings among followers such as higher job satisfaction (Cicero et al., 2010). In addition, a meta-analysis by Mathieu and Zajac (1990) found that a leader who initiates structure also increases organizational commitment among his followers. Organizational commitment refers to a psychological relationship an individual develops with an organization (Nascimento et al., 2008), both for emotional reasons and a moral obligation to stay (Meyer et al., 1993), giving individuals a sense of belonging within an organization (Nascimento et al., 2008). Therefore, we expect that IL will significantly predict organizational commitment.

### Team Identification

Prior research suggests that IL, especially leader prototypicality, increases team identification (TI; Hogg and van Knippenberg, 2003) a term which refers to a feeling of identification within a group and is often expressed by an individual seeing himself with similar characteristics to other members of the group (Dutton et al., 1994). Furthermore, TI has been found to be highly positively correlated with group commitment (Wann and Pierce, 2003). Also, identifying with a collective is proposed to lead to an increase in organizational commitment (Meyer et al., 2006; Johnson and Yang, 2010). Finally, Zhu et al. (2013) found that TI fully mediated the positive effect of transformational leadership on affective organizational commitment. We suspect that the positive effect of IL on organizational commitment is partly explained by how much the individual identifies with the team and thus that TI partially mediates the relationship between IL and organizational commitment.

### Participation in Decision Making

Team identification is probably not the only concept that explains the relationship between IL and organizational commitment. A leader who is “one of us,” who is “doing it for us,” who is “crafting a sense of us” and who is “making us matter” (Haslam et al., 2011), will likely facilitate the willingness of group members to participate in decision making. When there is a shared sense of social identity, the leader might create more opportunities for the members to participate and group members might be more willing to participate. Participation in decision making (PDM) can be conceptualized as a process of decision making that includes various parties (Knoop, 1995; Witt et al., 2000): from a decision made by one person to a combined group decision. PDM allows people to have more control over their work and environment (Witt et al., 2000) and results in both higher job satisfaction (Witt et al., 2000; Scott-Ladd et al., 2006) and greater work commitment (Mathieu and Zajac, 1990; Knoop, 1995; Scott-Ladd et al., 2006). Mathieu and Zajac (1990) found in their meta-analysis that leader communication and participative leadership were strongly correlated with organizational commitment. Thus, we expect that IL predicts PDM and that PDM predicts organizational commitment. We also expect that the effect of IL on organizational commitment is partially explained by perceived PDM.

### Collective Efficacy

While we are expecting that the relationship between IL and organizational commitment is partially mediated by PDM, this may not be the case for all workers in all teams. This mediation may be influenced by the level of team efficacy perceived by the team members. Self-efficacy is refers to one’s own belief that he is capable of producing certain effects through his actions (Bandura, 1998). When acting as a group, human agency is complemented by collective agency. Thus, collective efficacy (CE) is about shared beliefs in a group’s collective power and an emerging group-property rather than just the sum of the individual self-efficacy beliefs (Bandura, 1997). We suspect that people who are given the opportunity of applying shared decision making within their group decision profit more greatly when they believe that the CE of their group is high. Hence, we suspect that the positive relationship between PDM and organizational commitment will be moderated by CE. In addition, we suspect that people will strive for more participation if they perceive themselves as being capable of succeeding as a team. Therefore, we propose that CE also moderates the relationship between IL and PDM.

### Culture

For different cultures, working as a group has a different value. The Hofstede study (Hofstede, 2001) measures a global orientation toward the individual and its own interests or the collective (individualism/collectivism). Considering that the IL theory is based on social identity and group processes, and that different cultures behave differently within groups (House et al., 2004), we decided to investigate whether the model is generalizable across cultures that vary in terms of individualism-collectivism values by using two clusters of countries: one composed of individualistic countries and the other composed of collectivistic countries. In a large research study, van Dick et al. (2018) analyzed the generalizability of the IL model across 20 countries. Although those countries varied in terms of individualism-collectivism, the IL model was generalizable among all of them except Nepal. In all countries, IL predicts various outcomes such as job satisfaction, burnout, or organizational citizenship behavior. Furthermore, efficacy beliefs work cross-culturally, in individualistic as well as collectivistic cultures (Earley, 1994). Hence, we hypothesize that the model is generalizable across individualistic and collectivistic cultures.

### Aims and Hypotheses

To summarize, IL is a promising new approach in leadership research. Having a leader who creates and fosters a shared identity in the team leads to positive outcomes in the workplace such as higher job satisfaction (Cicero et al., 2010), lower turnover intentions by followers (Steffens and Haslam, 2013), and it may also increase organizational commitment. We propose that one way in which IL leads to increased organizational commitment is by increasing the individual identification with the team and by facilitating the willingness of group members to participate in decision making. These relationships, however, may be moderated and influenced by factors like perceived CE, and the individualism-collectivism values in a country. This leads to the following hypotheses.

**FIGURE 1:**
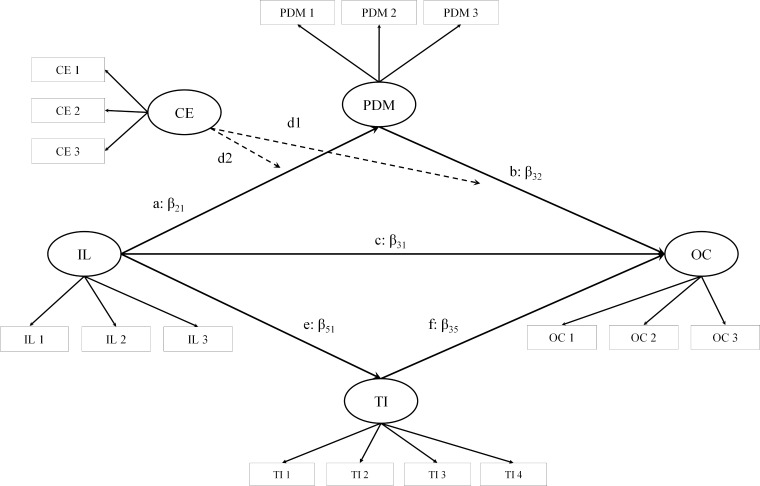
Hypothesized model.

In Study 1, we propose a model (see **Figure 1**) in which IL positively influences organizational commitment (OC; H1). We hypothesize that the impact of IL on OC is mediated by TI and PDM. A higher level of IL will lead to both a higher level of TI (H2) and a higher level of PDM (H3). Moreover, we expect that a higher level of TI (H4) and PDM (H5) will increase OC. Furthermore, we hypothesize that the relationship between IL and PDM (H6) as well as PDM and OC (H7) are moderated by CE. Finally, we suppose that this model is generalizable across individualistic and collectivistic cultures (H8). In Study 2, we will use an experimental design and focus on the causal relationship between IL and PDM. We hypothesize that there is a bidirectional causal relationship between IL and PDM, i.e., a leader who creates a shared sense of identity will make his subordinates more willing to participate in decisions (H9) and, in turn, greater PDM will increase the sense of shared identity created by the leader (H10).

## Study 1

### Method

#### Participants

Participants in Study 1 will be recruited from two clusters of countries according to their individualistic-collectivistic orientation. We used the measure of individualism/collectivism defined by the Hofstede study (Hofstede, 2001) which measures a global orientation toward the individual and its own interests or the collective. The scores range from 1 (collectivistic) to 100 (individualistic). In order to construct our two clusters, we used 50 as a cut-off point (Hofstede, 2001). Therefore, the collectivist cluster is comprised of Bulgaria (30), Portugal (27), and Turkey (37) and the individualist cluster is composed of France (71), Germany (67), Netherlands (80), Poland (60), and United Kingdom (89).

Based on our hypothesized model depicted in **Figure 1**, we will need to estimate 43 parameters (20 error variances, 11 factor loadings, five variances, seven regression path coefficients) which yields an estimated sample size of 430 per country cluster when 10 observations per estimated parameter are used (Bentler and Chou, 1987; Bollen, 1989). Due to the explorative nature of this study, we have no clear idea about the majority of effect sizes in the model. Therefore, we used this rule of thumb estimation of sample size over the more sophisticated Monte Carlo estimates.

There are three inclusion criteria: (1) participants should work in an organization, (2) have a direct supervisor, and (3) be part of a team of at least three people. Participation will be anonymous and voluntary.

#### Measures

##### Leadership

Participants will be asked to evaluate how their current supervisor/manager at work scores on each of the four dimensions of the IL by completing the IL Inventory (Steffens et al., 2014). The inventory is a 15-item questionnaire reflecting the four dimensions of the IL theory: identity prototypicality (e.g., “this leader embodies what the group stands for”), identity advancement (e.g., “this leader stands up for the group”), identity entrepreneurship (e.g., “this leader makes people feel as if they are part of the same group”), and identity impresarioship (e.g., “this leader devises activities that bring the group together”). Items will be rated on a seven-point Likert scale (1 = *not at all*; 7 = *completely*). Cronbach’s α varied from 0.88 to 0.92 (Steffens et al., 2014).

##### Organizational commitment

Participants’ organizational commitment will be measured by the 18-item scale developed by Meyer et al. (1993). The inventory measures three dimensions of organizational commitment: affective, continuance, and normative commitment. These three dimensions reflect the personal desire of respondents to stay in the organization (e.g., “I would be very happy to spend the rest of my career with this organization”), necessity to stay (e.g., “right now, staying with my organization is a matter of necessity as much as desire”), and loyalty to the organization (e.g., “I would feel guilty if I left my organization now”). Respondents rated items on a seven-point Likert scale (1 = *strongly disagree*; 7 = *strongly agree*). Cronbach’s α ranged from 0.77 to 0.85 (Meyer et al., 1993).

##### Team identification

Respondents will be administered the group identification measure (Doosje et al., 1995) in which four items regarding one’s group identification (e.g., “I identify with the other team members”) will be rated on a seven-point Likert scale (1 = *not at all*; 7 = *extremely*). For the aim of this study, the items will be adapted for an organizational context. The scale has a good reliability, Cronbach α = 0.83 (Doosje et al., 1995).

#### Participation in Decision Making

Participants will be asked to complete a group adapted version of the PDM scale (Witt et al., 2000). It is a six-item inventory which asks respondents to indicate how they and their managers make decisions in various contexts such as work appraisal. Answers will be scored on a five-point scale (1 = *we discuss things in a great detail and come to a decision based on consensus regarding the issue*; 2 = *we discuss things in a great deal and his/her decision is usually adopted*; 3 = *we discuss things in a great deal and the group decision is usually adopted*; 4 = *we don’t discuss things very much as his/her decisions are usually adopted*; and 5 = *we don’t discuss things very much and the group make most of the decisions*). The scale is reported to have good reliability, Cronbach α = 0.90 (Witt et al., 2000).

##### Collective efficacy

Participant will complete the seven-item CE Beliefs scale (Riggs et al., 1994) which measures CE in an organizational setting. On a seven-point Likert scale, respondents would rate items such as (“the team I work with has above average ability”). The scale is reported to have good reliability, Cronbach α = 0.88 (Riggs et al., 1994).

##### Socio-demographic information

Participants will be asked to provide socio-demographic information (sex, age, nationality, and education), as well as information regarding their job: work field, type of employment (e.g., full time or part time), type of contract (temporary or permanent), country in which they are working, team size, and number of years spent working in their team.

#### Procedure

The materials will be translated into the languages of the targeted countries. The questionnaires (i.e., IL, PDM, OC, CE, and TI) which did not previously exist in the target languages (Bulgarian, Dutch, French, German, Polish, Portuguese, and Turkish) will be translated into the respective languages following the back-translation technique (Brislin, 1970). The IL measure was already validated in Dutch, French, German, and Turkish (van Dick et al., 2018). The adapted versions of OC in Dutch, French, German, Polish, Portuguese, and Turkish were already validated (de Gilder et al., 1997; Vandenberghe et al., 2001; Wasti, 2002; Süß, 2007; Nascimento et al., 2008; Bañka and Wooska, 2015); the Bulgarian version of OC will be translated with the back-translation technique. Furthermore, PDM, TI, and CE will need to be translated in all targeted languages. The factor structure of all translated measures will be assessed.

Data will be collected using an online questionnaire on Qualtrics. Participants will be recruited using the snowball sampling technique: via email, social media, personal contact, and work environment. Participation will be anonymous and voluntary. After giving their informed consent, participants will answer three inclusion criteria questions (e.g., “are you currently working as an employee?”). Those who correspond to our target population will then have to answer the questionnaire including measures of the IL, PDM, CE, organizational commitment, TI, and the socio-demographic information. The presentation order of the measures will be randomized, in order to avoid any order effects.

#### Planned Analysis

We will report all data exclusions (if any), all manipulations, and all measures in the studies. All analyses will be done with the GNU R software (R Core Team, 2013).

##### Moderation and mediation effects based on entire sample

To investigate the underlying relationships between IL and OC as well as the potential mediation of TI and PDM and the moderation effect of CE, we will first conduct a structural equation modeling analysis based on the entire sample. The hypothesized model is depicted in **Figure 1**.The latent variables IL, OC, PDM, and CE are defined by three indicators each. These indicators will be generated by item parceling. Parceling is when items are combined (summed or averaged) prior to an analysis and the parcels (instead of the original items) are used as the manifest indicators of latent constructs (Cattell, 1956). Instead of parcels, for TI, we will use the four corresponding items as indicators. As shown in **Figure 1**, we hypothesize that the relationship between IL and OC is partially mediated by TI. Furthermore, PDM partially mediates the relationship between IL and OC. In addition, CE potentially moderates both the relationship between IL and PDM as well as the relationship between PDM and OC. For the analyses, we will use the lavaan package in R (Rosseel, 2012). Before the actual analyses, we will evaluate the univariate normality assumption by examining skewness and kurtosis using the psych package (Revelle, 2017). Absolute values of skewness and kurtosis < 1 implicate univariate normality (Kline, 2011). We also will assess the multivariate normality assumption with Mardia’s multivariate test (Mardia, 1970) by using the MVN package (Korkmaz et al., 2014).

###### Model fit

We will follow the recommendations from Kline (2011) and Schermelleh-Engel et al. (2003) and use several fit indices to interpret the model fit in general. First, we will use Chi-square (χ^2^) and its associated p value, χ^2^/df. Because χ^2^ is sensitive to sample size and the violation of the multivariate normality assumption, we will also include different classes of goodness-of-fit criteria: the root-mean-square error of approximation (RMSEA; Steiger, 1990), the comparative fit index (CFI; Bentler, 1990), the standardized root-mean-square residual (SRMR; Jöreskog and Sörbom, 1989), and the Tucker–Lewis index (TLI; Tucker and Lewis, 1973). As recommended by Chen et al. (2008) as well as Marsh et al. (2004a), we will interpret the global model fit based on the constellation of these indices.

###### Mediation

To investigate the mediation effect, we will use bias-corrected bootstrapping to estimate confidence intervals for the indirect effect based on the recommendation of MacKinnon et al. (2007) and results of MacKinnon et al. (2004) and Fritz and MacKinnon (2007). This will allow us to test H2–H5 and to study if the relationship between IL is mediated by TI and PDM. In addition, we will use the ratio of indirect effect to total effect (Wen and Fan, 2015) as additional indicator.

###### Moderation

Although interaction or moderation effects are common in social sciences, estimating such effects in SEM, however, is difficult and not straightforward. A plethora of competing strategies and statistical approaches have been proposed (see, e.g., Jaccard and Wan, 1995; Jöreskog and Yang, 1996; Algina and Moulder, 2001; Marsh et al., 2004b; Little et al., 2006), which are mostly based on the product indicator model from Kenny and Judd (1984). We will use the double-mean-centering approach proposed by Lin et al. (2010) which is identical or superior to the single-mean-centering (Marsh et al., 2004b) and orthogonalizing strategies (Little et al., 2006) that have been proposed previously. Thus, we can test H6 and H7 and explore if CE moderates the relationship between IL and PDM as well as PDM and OC.

##### Differences between groups

We will use multiple-group analyses to explore and test H8, i.e., whether differences in the structural parameters across groups of individualistic and collectivistic countries were statistically significant. To test for group invariance, we will compare two nested models with a likelihood ratio test (Bentler and Bonett, 1980; Bollen, 1989; Ryu, 2015). First, we will compare a baseline model wherein no constraints were specified and a second model where all factor loadings were constrained to be invariant between the groups. In the next step, we will compare this model with a model where all path coefficients were constrained to be invariant between the groups. In addition, when there are differences between the unconstrained and constrained models, subsequent likelihood ratio tests will be conducted, where different paths will be constrained and tested against the unconstrained model.

### Anticipated Results

A leader who creates a shared sense of identity can lead to more commitment to the organization. We therefore expect to find a positive relationship between IL and OC. Based on previous research studies, we hypothesize that TI mediates this relationship. We further hypothesize that PDM is mediating the relationship between IL and OC, because when there is a shared sense of social identity, the leader might create more opportunities for the members to participate and group members might be more willing to participate. Additionally, CE is supposed to act as a moderator on this mediation. This is proposed to happen in such a way that the positive effect of IL on OC mediated by PDM is higher for participants with higher CE. Finally, we propose this as a cross-cultural model which is generalizable across individualistic and collectivistic cultures.

Depending on which hypotheses are supported by the results, different pieces of advice could be given to developing or established leaders in organizations. When there is a positive relationship between IL and OC, one might argue that leaders and organizations will profit from adopting IL behaviors, since OC leads to beneficial outcomes such as higher performance and employee well-being (Kurtessis et al., 2017). A possible mediation of the effect of IL on OC by TI and PDM would suggest that if a leader is wondering how to best foster organizational commitment in his followers, we would advise them to focus on the following: creating opportunities for followers to be actively involved and able to participate in decision making. Furthermore, a leader might strengthen OC by promoting identification within the group. Also if we find that CE is moderating the mediation of PDM, we would advise leaders that when attempting to increase OC by letting their followers participate in decision making, they should make sure that members of the team perceive their group as competent and effective when dealing with challenges and coming to a decision together.

It is important to identify the potential mediators and moderators of the relationship between IL and work commitment. This would help to better understand the impact of IL, to explore the way it works and to design programs that maximize its impact on organizational commitment and other organizational outcomes.

### Anticipated Limitations

There are certain limitations of this study that future research should focus on. First, the clusters of individualistic and collectivistic countries that will be tested include only European countries and Turkey, thus sampling cultures from other continents can be potentially helpful to improve our understanding. Additionally, although the study will provide significant insight into this research question, the correlational nature of the study is problematic for the internal validity of this piece of research. Based on the literature, we assumed that IL of a leader will predict the level of PDM among his followers. However, it is also possible that followers who have a higher level of PDM will be more likely to perceive their leader as an identity leader than those who have a lower level of PDM. The direction of this relationship is neither clear in the literature nor based on our first study. Therefore, the second study will address this issue by using an experimental design which will provide in-depth understanding of the relationship between IL and PDM. By manipulating the degree of IL and PDM, we will aim to establish whether scoring high on IL encourages PDM or PDM shapes one’s perception of the leader as creating a shared sense of identity.

## Study 2

### Overview

In Study 1, we studied the correlational relationship of IL, OC, TI, PDM, and CE within two country clusters. To further investigate the direction of the underlying causal processes, in Study 2, we will use an experimental design and focus on the causal relationship between IL and PDM. We hypothesize that there is a bidirectional causal relationship between IL and PDM, i.e., (1) a leader who creates a shared sense of identity will make his subordinates more willing to participate in decisions (H9) and (2) greater PDM will increase the perception of shared identity created by the leader (H10).

### Method

#### Participants

An *a priori* sample size calculation with G-Power 3.1 (Faul et al., 2007) showed that for six conditions of IL manipulation, 36 participants per condition are required to achieve a power of 0.80 with α = 0.05 and an expected medium effect size, *f* = 0.25. In addition, for the two conditions of manipulation of the level of PDM, the sample size estimate resulted in 64 participants per group, based on α = 0.05, a power of 0.80, and an expected medium effect size, *d* = 0.5. Therefore, this study aims to recruit 344 participants. Participants will be recruited in English speaking countries using the snowball sampling technique: via email, social media, personal contact, and work environment. There are three inclusion criteria: (1) participants should work in an organization, (2) have a leader, and (3) be part of a team of at least three people. Participation will be anonymous and voluntary.

#### Materials and Procedure

After giving their informed consent, participants will answer the same three inclusion criteria questions as used in Study 1. Those who correspond to our target population will be randomly assigned to one of eight possible conditions. Six of these conditions are dedicated to manipulate IL, the other two manipulate one’s level of participation in the decision making process at work. In regard of IL manipulation, participants will be presented with a short description of the behavior of a manager in the workplace based on the IL model. In every description, each of the four dimensions of IL will be manipulated to be either in the high or in the low version (e.g., “your manager exemplifies/does not exemplify what it means to a member of this group”). Thus, there will be six possible versions: one with all dimensions being in the high version, one with all dimensions being in the low version, and four more in which the hypothetical manager would score high on one dimension but low on the other three dimensions. In the conditions dedicated to manipulating PDM, participants would read a paragraph in which one would be either highly involved in discussions and the decision making process (e.g., “your supervisor listens to each and every one of you and you, all together, come to a decision that everybody agrees to”) or barely involved in the decision making process (e.g., “your supervisor comes to most of the decisions, without considering what you have to say”). All manipulations (six IL and two PDM) will be pre-tested in an online survey. After reading the manipulation, participants will complete the two scales measuring IL (Steffens et al., 2014) and PDM (Witt et al., 2000). These two measures are described in section “Study 1.” For the six manipulation of IL conditions, participants will answer the PDM first and then the IL (which will be used as a manipulation check). For the manipulation of the two PDM conditions, they will answer the IL first and then the PDM (which will be used as a manipulation check). At the end of the study, participants will have to provide the same socio-demographic information as in Study 1.

#### Planned Analysis

R (R Core Team, 2013) will be employed to investigate the direction of the relationship between IL and PDM with an online experiment.

##### Identity leadership

In order to examine how IL, defined as four dimensions by Haslam et al. (2011), affects the PDM processes of group members (H9), a one-way ANOVA will be performed. The manipulation of IL will result in six conditions: the presentation of the leader will be either (1) high on the four dimensions, or (2) low on the four dimensions, or high on one of the dimensions and low on the other – (3) prototypicality dimension is high – rest is low, (4) advancement dimension is high – rest is low, (5) entrepreneurship dimension is high – rest is low, and (6) impresarioship dimension is high – rest is low. Thus, we will observe the effect of the six different descriptions of the leader on PDM. First, we will test the normality of the distribution of the residuals by analyzing the skewness and kurtosis as well as using residuals vs. fitted and normal QQ plots (Hwu et al., 2002; Field, 2013). Afterward, the Levene test will be employed to assess the homogeneity of variances (Levene, 1960). If the normality of the distribution and homogeneity of variances are confirmed, a one-way ANOVA with planned contrast will be performed to compare any differences between the six groups (Norusis, 2008). First, we will compare the high on all dimension condition with the five other conditions (1 vs. 2, 3, 4, 5, 6) and then we will compare the low on all dimension condition with the four other conditions (2 vs. 3, 4, 5, 6). As we do not have specific hypotheses regarding the four conditions in which IL is high on one dimension and low on the three others, we will use *post hoc* tests following the guidelines of Field (2013) to compare the differences between these four groups.

##### Participation in decision making

A *t*-test will be performed in order to detect if the level of PDM of team members has an effect on the perception of IL (H10). After checking the assumptions (normality, homogeneity of variances), we will compare the level of perceived IL between the low and high PDM conditions using an independent-samples *t*-test. Lastly, we plan to report the 95% confidence interval (Coe, 2002) and effect size using cohen’s *d* (Cohen, 1988).

### Anticipated Results

In Study 2, we manipulate the degree of IL practiced by a leader in six conditions. We anticipate that a leader with a high degree of IL in every dimension of IL will make team members want to participate more in decision making compared to a leader with a medium or low degree of IL. In addition, we also anticipate that by manipulating the amount of PDM in a group, the leader will appear more as creating and fostering a shared identity in his team. This would allow us to formulate practical guidelines for increasing the organizational commitment of workers.

### Anticipated Limitations

This piece of research will advance our understanding of the relationship between IL and PDM and may contribute to the way managers approach decision making with their employees. Investigating the way in which one’s level of PDM shapes the image of their manager is particularly important, as managers can use PDM as a tool to enhance the shared sense of identity amongst the team. There are, however, certain limitations with regard to the generalizability of the results that future research should address. Conducting the experiment through an online platform can potentially influence responses and future research should aim to test the model in more realistic settings. Additionally, a more complex experimental design may want to establish additional relationships by including variables such as TI and CE.

## Conclusion

Even though IL is still in its infancy, numerous studies have suggested its importance in predicting positive work related outcomes (e.g., Cicero et al., 2010; Hogg et al., 2012). The goal of our research study is to identify how a supervisor who adopts behaviors based on the IL principles can increase commitment in the organization among his followers. Therefore, this study may help to provide practical guidelines for supervisors as a way to increase commitment. Depending on the results, we could advise leaders to focus on promoting identification of their followers within their team and providing their followers with choices and opportunities to participate in decision making. Regarding the latter, we would recommend that leaders improve the perceptions of collective-efficacy held by their followers (e.g., through creating success stories, team encouragement, and the promotion of in-group collaboration (Bandura, 1998; Goddard et al., 2007), with the purpose of moderating the positive relationship toward organizational commitment. The model is not restricted for application in the work environment, but can also be transferred to other non-organizational contexts such as education, sports, politics or NGOs.

## Ethics Statement

This study will be carried out in accordance with the recommendations of “Comité d’Ethique Interne du laboratoire C2S” with written informed consent from all subjects. All subjects will give written informed consent in accordance with the Declaration of Helsinki. The protocol was approved by the “Comité d’Ethique Interne du laboratoire C2S.”

## Author Contributions

The initial design of this study was conceptualized by JL who also supervised the project and provided feedback. The rest of the authors also contributed to refining the design upon the start of their team work. MH, AK, and RK were in charge of the method section. MM and FD worked on the introduction section. The planned analysis section was produced by DI and AT.

## Conflict of Interest Statement

The authors declare that the research was conducted in the absence of any commercial or financial relationships that could be construed as a potential conflict of interest.
